# TrenibotulinumtoxinE: Pharmacology, clinical evidence, and emerging role in aesthetic medicine

**DOI:** 10.1016/j.jpra.2026.09.019

**Published:** 2026-09-18

**Authors:** Chaocheng Liu, Jean Carruthers, Alain Michon

**Affiliations:** a Department of Dermatology and Skin Science, University of British Columbia, Vancouver, BC, Canada; b School of Medicine, Simon Fraser University, Surrey, British Columbia, Canada; c Department of Ophthalmology, University of British Columbia, Vancouver, BC, Canada; d Jean Carruthers Cosmetic, Vancouver, BC, Canada; e Project Skin MD, Ottawa, ON, Canada

**Keywords:** TrenibotulinumtoxinE, Botulinum neurotoxin type E, Glabellar lines, Facial aesthetics, Neuromodulator

## Abstract

Botulinum neurotoxin type A (BoNT/A) products are among the most popular aesthetic treatments worldwide, but their relatively prolonged duration may be a barrier for patients who are concerned about unnatural appearance, adverse effects, or being committed to an unwanted result. TrenibotulinumtoxinE (trenibotE), a botulinum neurotoxin type E formulation, has a distinctly rapid onset and shorter duration of action, providing a pharmacologic profile that differs from currently available BoNT/A products and may better meet the needs of certain patients seeking greater treatment flexibility. This narrative review summarizes the pharmacology, clinical development, efficacy, safety, and potential aesthetic role of trenibotE based on preclinical and clinical studies. Preclinical studies demonstrate a more rapid onset and shorter duration of neuromuscular effect with trenibotE than with BoNT/A, likely reflecting differences in intracellular persistence, receptor interactions, and SNAP-25 cleavage. In clinical studies of moderate-to-severe glabellar lines (GL), improvement was evident within 8–24 hours, with peak efficacy around Day 7 and return toward baseline by approximately Day 21. Phase 3 trials reported that approximately 60%–66% of trenibotE-treated participants achieved a ≥ 2-grade improvement to none or mild GL severity at Day 7. Patient-reported outcomes showed high satisfaction, including satisfaction with natural-looking results and improvement in the psychological impact of GL. TrenibotE was generally well tolerated, with low rates of treatment-related adverse events and no treatment-emergent neutralizing antibodies reported. TrenibotE represents a rapid-onset, short-acting neuromodulator with a clinical profile that may complement existing BoNT/A treatments. Its shorter duration may be particularly appealing to toxin-naive or treatment-hesitant patients and may provide greater flexibility for individualized aesthetic treatment. Further studies are needed to characterize repeated use, long-term safety and immunogenicity, diffusion, dose optimization, treatment of non-glabellar areas, and direct comparisons with BoNT/A.

## Introduction

There are seven antigenically distinct classical serotypes of botulinum neurotoxin (BoNT), designated A through G.^1^ Although produced by different strains of *Clostridium botulinum*, these serotypes share a broadly similar structure and mechanism of action. Each serotype cleaves a distinct site on one or more intracellular proteins involved in synaptic vesicle release, thereby inhibiting acetylcholine release from presynaptic terminals at the neuromuscular junction and producing temporary skeletal muscle weakness.

Multiple botulinum neurotoxin serotype A (BoNT/A) products are currently approved for aesthetic treatment of dynamic facial concerns, including glabellar lines (GL), lateral canthal lines, forehead lines, and, in some jurisdictions, masseter hypertrophy. In clinical practice, off-label treatment of other facial muscles is also common as part of a more comprehensive approach to facial rejuvenation.

Despite the generally favorable safety profile of neuromodulators, some patients remain hesitant to pursue treatment because of concerns about an unnatural appearance, unsatisfactory outcomes, or adverse effects, as reported in a global survey of 14,584 adults.^2^ From the provider perspective, a survey of 141 aesthetic practitioners found that concern about a frozen or unnatural appearance was reported at least sometimes in 86.6% of responses regarding younger toxin-naïve patients and in 93.0% regarding older patients.^3^ These findings reinforce the importance of treatment-related appearance concerns in clinical decision-making, although preferences for a shorter-duration treatment were not directly assessed. Consumer market research involving 12,286 adults across nine countries found that 79% wished they could temporarily preview the outcome of an aesthetic treatment, suggesting substantial interest in the ability to experience or assess an aesthetic result before committing to a longer-lasting intervention.^4^

Collectively, these findings provide a rationale for neuromodulators with a rapid onset and shorter duration of effect, which may help address some of these barriers by offering a more temporary and potentially less committing treatment option. TrenibotulinumtoxinE (trenibotE) is the first-in-class botulinum toxin serotype E. This review summarizes the biologic and pharmacologic characteristics of trenibotE, its clinical efficacy and safety, and its potential role in aesthetic practice.

### MECHANISM OF ACTION AND PHARMACOLOGY

BoNT/E binds to receptors on the presynaptic nerve terminal and is internalized into synaptic vesicles through receptor-mediated endocytosis. Following vesicle entry, the toxin light chain translocates into the cytosol, where it proteolytically cleaves synaptosomal-associated protein of 25 kDa (SNAP-25), a key component of the soluble N-ethylmaleimide-sensitive factor attachment protein receptor (SNARE) complex required for synaptic vesicle fusion, thereby inhibiting neurotransmitter release.^5^^,^^6^

The pharmacologic activity of trenibotE has been characterized using the mouse Digit Abduction Score (DAS) assay. Following intramuscular administration to hindlimb skeletal muscle at doses ranging from 18.2 to 182 U/kg, trenibotE produced dose-dependent increases in peak DAS response and duration of effect.^7^^,^^8^ Onset of activity was observed by 6 h at the two lowest doses and as early as 3 h, the earliest assessment time point, at doses of 45.5 U/kg and above. Peak effect occurred within approximately 12–27 h, and recovery was relatively rapid: responses returned to baseline within 30–48 h at lower doses and by approximately 72 h at higher doses.^7^^,^^8^ These findings establish a rapid, dose-dependent, and short-lived pharmacodynamic profile for trenibotE.

This temporal profile differs substantially from that of BoNT/A and appears to reflect several intrinsic biologic and pharmacologic differences between the two serotypes. Although both inhibit acetylcholine release through SNAP-25 cleavage, their intracellular persistence, structural configuration, receptor interactions, and cleavage sites differ.

One important distinction is the shorter intraneuronal persistence of the BoNT/E light chain.^9^ BoNT/E is predominantly localized within the cytosol, where it may be more readily degraded.^10^ In contrast, the BoNT/A light chain associates with the plasma membrane and slow-recycling intracellular compartments and interacts with the deubiquitinating enzyme VCIP135, mechanisms that may protect it from proteasomal degradation and prolong intracellular activity.^11^^,^^12^

The more rapid onset of BoNT/E may also relate to its structural organization. Whereas the binding, translocation, and catalytic domains of BoNT/A adopt a more linear or “open” configuration, BoNT/E has a more compact “closed” conformation. This arrangement may facilitate faster translocation of the BoNT/E light chain across the endosomal membrane and thereby contribute to its more rapid onset of action.^13^^,^^14^^,^^15^

BoNT/E and BoNT/A also differ in their recognition of synaptic vesicle protein 2 (SV2) isoforms. BoNT/E preferentially binds glycosylated SV2A and SV2B, whereas BoNT/A can bind SV2A, SV2B, and SV2C, with a preference for SV2C.^14^ Differences in the expression and tissue distribution of these isoforms may therefore contribute to variation in biologic activity, time course of effect, and unit potency between the two serotypes.

A further distinction lies in the site of SNAP-25 cleavage. BoNT/E cleaves SNAP-25 at residue 180, removing 26 amino acids from the C-terminus, whereas BoNT/A cleaves at residue 197, removing only the terminal 9 amino acids.^5^^,^^6^^,^^16^ The more extensively truncated SNAP-25 fragment generated by BoNT/E is less able to participate in functional SNARE-complex assembly and support synaptic vesicle fusion. It may also be less stable within the synaptic terminal, allowing more rapid degradation, earlier replenishment of functional SNAP-25, and faster restoration of neurotransmission, thereby contributing to the shorter duration of BoNT/E activity.^17^^,^^18^

These mechanistic differences are reflected in preclinical pharmacodynamic comparisons. In the mouse DAS assay, trenibotE at approximately equi-efficacious doses demonstrated a more rapid onset and substantially shorter duration of effect than historical onabotA controls.^7^^,^^8^ TrenibotE reached peak effect within approximately 12–27 h, compared with 48–72 h for onabotA, and although both showed activity as early as 6 h, trenibotE produced significantly greater DAS responses between 6 and 27 h. At approximately equi-efficacious doses within the ED75–ED90 range (i.e., doses producing 75%–90% of maximal effect), neuromuscular function returned to baseline by approximately 72 hours with trenibotE versus 2–3 weeks with onabotA.^7^

Other potential distinctions include differences in intramuscular diffusion and local spread; however, the clinical relevance of these findings remains incompletely defined and requires further direct comparative study.^19^^,^^20^ Overall, the available evidence suggests that the rapid onset and short duration of trenibotE arise from multiple interacting mechanisms rather than a single pharmacologic feature. In addition, trenibotulinumtoxinE potency units are product-specific and should not be directly compared with or converted to units of botulinum toxin type A formulations.

### Clinical development

In a first-in-human, randomized, double-blind, placebo-controlled, ascending-dose Phase 2 study, 42 participants with GL received EB-001 (BoNT/E; n = 35) or placebo (n = 7) (Table 1).^21^ The primary efficacy endpoint was a ≥ 2-grade improvement from baseline in investigator-rated Facial Wrinkle Scale (FWS) severity at maximum frown. Responses emerged in the third dose cohort and increased with dose, reaching 80% at the highest doses.^21^ Clinical effect was evident within 24 h, with duration ranging from approximately 14 to 30 days at the highest doses.^21^ EB-001 was generally well tolerated; headache was the most common adverse event, with no serious adverse events or ptosis reported.^21^

**Table 1:** Summary of clinical studies evaluating trenibotulinumtoxine for glabellar lines.

Outcome / characteristic	Yoelin 2018	Lain & Baumann 2026	Lain & George 2026	Moers-Carpi 2026	Hanke 2026
**Phase / design**	Phase 2, randomized, double-blind, placebo-controlled, ascending-dose cohort study	Phase 2b, multicenter, double-blind, randomized, placebo-controlled, dose-escalation study	Phase 3, multicenter, double-blind, randomized, placebo-controlled study; toxin-naive population	Phase 3, double-blind, placebo-controlled study; randomized 3:1; DB + optional OL retreatment	Phase 1, multicenter, randomized study; DB period followed by OL onabotA
**Study duration**	42 days (approximately 6 weeks)	42 days	12 weeks	12 weeks	5 months
**Completed, n (%)**	41 (97.6%)	190 (96.0%)	274 (88.7%), DB period	89.2%	83 (92.2%)
**Female, n (%)**	EB-001 32 (91.4); placebo 6 (85.7)	182 (91.9), pooled	245 (79.3), pooled	88.7%, pooled	79%, pooled
**White, n (%)**	EB-001 25 (71.4); placebo 5 (71.4)	175 (88.4), pooled	257 (83.2), pooled	90.0%, pooled	92%, pooled
**Age, mean, y (SD or range)**	EB-001 47.9 ^22^-^60^; placebo 50.4 (32-57)	48.3 (9.91), pooled	43.1 (13.08), pooled	47.2, pooled	49, pooled
**Participants randomized, n**	42 (EB-001 35; placebo 7)	198 (trenibotE 148; placebo 50)	309 (trenibotE 227; placebo 82)	638	90 (trenibotE 48; placebo 42)
**Baseline investigator-rated FWS: moderate / severe, n (%)**	NR	83 (41.9) / 115 (58.1), pooled	85 (27.5) / 224 (72.5), pooled	NR	Severe: 75.6%, pooled
**Baseline FLO-11 total score, n (%)**	NR	NR	≤50: 227 (73.5); >50: 82 (26.5)	NR	NR
**Treatment regimen**	Seven ascending-dose cohorts; EB-001 or placebo (5:1 per cohort); 5 IM injections, 0.1 mL/site (procerus + bilateral medial/lateral corrugators); total 0.5 mL	Five dose cohorts; trenibotE 100-750 U total or placebo (3:1); 20-150 U/0.1 mL; 5 IM injections (1 procerus, 2/corrugator), 0.1 mL/site	TrenibotE 700 U or placebo (3:1); 5 IM injections (1 procerus, 2/corrugator), 140 U/injection; total 0.5 mL	TrenibotE 700 U or placebo (3:1); eligible participants could receive OL retreatment	TrenibotE 700 U or placebo on Day 1; eligible participants received onabotA 20 U on Day 30
**Primary efficacy endpoint**	≥2-grade investigator-rated FWS improvement at maximum frown at any postbaseline visit through Day 30	Cumulative ≥2-grade investigator-rated FWS improvement at maximum frown through Day 7	Grade 0/1 plus ≥2-grade improvement at maximum frown on both investigator- and participant-rated FWS at Day 7	Grade 0/1 plus ≥2-grade improvement at maximum frown on both investigator- and participant-rated FWS at Day 7	Investigator-rated FWS grade None/Mild at Day 60 (30 days after onabotA)
**Key efficacy result**	80.0% in highest-dose cohorts vs 14.3% placebo	96.8% in highest-dose cohort (Cohort 5); participant-rated cumulative ≥2-grade response 71.0%	65.7% (95% CI, 59.5%−71.9%) vs 1.2% (95% CI, 0.0%−1.2%)	60.0% vs 0.6% at Day 7	75.0% (95% CI, 61.6%−88.4%) vs 80.6% (95% CI, 67.6%−93.5%)
**Onset / duration**	Clinical effect within 24 h; highest doses lasted approximately 14-30 days	Effect observed by Hour 8; clinically meaningful difference by Hour 24 in Cohort 5; persisted through Day 14 in Cohort 5	Effect observed at Hour 8; peak Day 7; returned to moderate/severe by Day 21	Effect observed at Hour 8; 30.4% vs 0.6% response at Hour 24; returned to baseline by Day 21	NR for trenibotE in this abstract; primary assessment after onabotA at Day 60
**Selected PROs**	NR	Cohort 5 showed greater satisfaction with natural look and overall effect and ≥20-point FLO-11 improvement vs placebo	Appearance-related psychological-impact subgroup: participant-rated primary-type response 67.9% vs 0%; Day-7 overall satisfaction 86.6% vs 6.3%; ≥20-point FLO-11 improvement 61.9% vs 7.9%	NR	NR
**TEAEs**	5/35 (14.3%) vs 0/7 (0%)	55/148 (37.2%) vs 21/50 (42.0%)	48/227 (21.1%) vs 19/82 (23.2%), DB period	NR	32.6% vs 33.3% during treatment period 2 (after onabotA)
**Treatment-related TEAEs**	NR	33/148 (22.3%) vs 10/50 (20.0%)	17/227 (7.5%) vs 7/82 (8.5%), DB period	7.7% vs 9.6%, DB period	NR
**TEAEs leading to study-drug discontinuation**	0 vs 0	0 vs 0	4/227 (1.8%) vs 0/82 (0%); all 4 events were unrelated to study treatment	NR	NR for study-drug discontinuation; 1 participant discontinued the study because of an AE unrelated to study treatment

Notes: Values are presented as reported in the five uploaded source publications/abstracts. “Pooled” denotes values reported for the entire study population rather than separately by treatment arm. For Moers-Carpi, the abstract reports 89.2% study completion without an absolute n. Hanke TEAE rates of 32.6% and 33.3% refer specifically to treatment period 2 after onabotA; the abstract reports one study discontinuation due to an AE unrelated to treatment, but does not identify it as a study-drug discontinuation.

**Abbreviations:** CI, confidence interval; DB, double-blind; FLO-11, Facial Line Outcomes 11; FWS, Facial Wrinkle Scale; IM, intramuscular; NR, not reported; OL, open-label; onabotA, onabotulinumtoxinA; SD, standard deviation; TEAE, treatment-emergent adverse event; trenibotE, trenibotulinumtoxinE.

A subsequent multicenter, double-blind, randomized, placebo-controlled, dose-escalation Phase 2b study evaluated trenibotE in 198 adults with moderate-to-severe GL in the United States, with follow-up through Day 42.^22^ Participants were randomized 3:1 to trenibotE or placebo, and 96% completed the study.^22^ Treatment was administered across five standard glabellar injection sites, including one injection into the procerus and two into each corrugator muscle. A clear dose-response relationship was observed. For the primary efficacy endpoint, defined as a cumulative ≥2-grade improvement from baseline on the investigator-rated FWS through Day 7, response rates increased from 40.6% in Cohort 1 (lowest dose) to 96.8% in Cohort 5 (highest dose) and were consistently higher than with placebo.^22^ Clinical activity was evident as early as 8 h after treatment, with meaningful improvements apparent by 24 h and persisting through Day 7 in Cohorts 1–4 and through Day 14 in Cohort 5.^22^ Participant-rated FWS responses showed a similar pattern, with 34.4% to 71.0% achieving a ≥ 2-grade improvement at maximum frown by Day 7.^22^ Patient-reported outcomes also supported the clinical findings. Participants in Cohort 5 reported high satisfaction with the natural appearance and overall treatment effect, together with meaningful reductions in the psychological impact of GL, reflected by a ≥ 20-point improvement from baseline in Facial Line Outcomes-11 (FLO-11) scores.^22^ Notably, satisfaction remained high through Day 42 despite investigator-rated severity generally returning to moderate or severe by Day 21, suggesting that perceived treatment benefits may persist beyond the period of objective FWS response.^22^

TrenibotE was well tolerated, with similar treatment-related adverse events (TEAEs) rates versus placebo (22.3% vs 20.0%) and no serious TEAEs, treatment discontinuations, or events suggestive of toxin spread or local diffusion.^22^ Collectively, these dose-ranging studies established a rapid, dose-dependent response and supported selection of the 700-U dose for subsequent Phase 3 development.

### Clinical efficacy and safety

Two Phase 3 trials evaluated the efficacy and safety of trenibotE for moderate-to-severe GL (Table 1).^23^^,^^24^ The first was a 12-week, double-blind, placebo-controlled study conducted in the United States, Canada, and the European Union.^23^ Among 638 randomized participants (88.7% female, 90.0% White; mean age, 47.2 years), participants were assigned 3:1 to trenibotE 700 U or placebo, and 89.2% completed the study.^23^ During the 6-week double-blind period, assessments were performed from Hour 8 through Day 43, followed by a 6-week open-label period.^23^ At Day 7, the primary endpoint was a ≥ 2-grade improvement from baseline to none or mild GL severity at maximum frown on both investigator- and participant-rated FWS, and it was achieved by 60.0% of trenibotE-treated participants compared with 0.6% receiving placebo.^23^ Treatment effect was evident as early as Hour 8, with 30.4% meeting response criteria by Hour 24 versus 0.6% with placebo. Efficacy peaked at Day 7 and declined toward baseline by Day 21.^23^ No treatment-related serious adverse events were reported.^23^

The second Phase 3 study evaluated 309 neurotoxin-naive adults with moderate-to-severe GL in the United States and Canada.^24^ Participants were randomized 3:1 to trenibotE 700 U or placebo; 79.3% were female, 83.2% were White, and the mean age was 43.1 years. At baseline, 27.5% had moderate and 72.5% had severe GL.^24^ Treatment was administered across five glabellar injection sites, with one injection into the procerus and two into each corrugator muscle. At Day 7, the primary endpoint of achieving none or mild GL severity together with a ≥ 2-grade improvement from baseline at maximum frown was achieved by 65.7% of trenibotE-treated participants versus 1.2% receiving placebo.^24^ Among participants with baseline appearance-related psychological impact, 67.9% achieved the corresponding participant-rated FWS endpoint, and 61.9% achieved a ≥ 20-point improvement in FLO-11 scores versus 7.9% with placebo.^24^ Patient satisfaction was also high, with 84.9% reporting that they were mostly or very satisfied with treatment and 84.7% satisfied with the natural-looking results.^24^ Consistent with the other Phase 3 trial, onset was rapid. At Hour 8, ≥1-grade FWS improvements were observed in 34.4% of participants by investigator assessment and 32.2% by participant assessment, increasing to 59.5% and 57.8%, respectively, by Hour 12 and approaching 90% by Hour 48.^24^ Treatment satisfaction also emerged early, increasing from 42.9% at Hour 8 to 69.3% at Hour 24. Clinical efficacy peaked at Day 7 and declined thereafter, with GL generally returning to moderate or severe by Day 21.^24^ Treatment-related adverse events occurred at similar rates with trenibotE and placebo (7.5% vs 8.5%), with no treatment-related serious adverse events reported.^24^

Given its shorter duration of action, trenibotE may serve as a lower-commitment initial treatment for patients hesitant to undergo longer-lasting neuromodulator therapy. Patients satisfied with treatment may subsequently transition to a longer-acting neuromodulator; however, the safety and efficacy of sequential administration of different BoNT serotypes had not previously been established. To address this, Hanke et al. conducted a multicenter, randomized, 5-month Phase 1 study evaluating trenibotE followed by onabotA in adults with moderate-to-severe GL.^25^ Ninety participants were randomized 1:1 to trenibotE 700 U or placebo on Day 1. Beginning on Day 30, participants whose GL severity had returned to moderate or severe received onabotA 20 U. At Day 60, the primary endpoint of none or mild GL severity on investigator-assessed FWS was achieved by 75.0% of participants previously treated with trenibotE and 80.6% of those previously receiving placebo.^25^ Sequential treatment was well tolerated, with no new safety signals and comparable response to onabotA regardless of prior trenibotE exposure.^25^ No treatment-emergent binding or neutralizing antibodies to either toxin was observed.^25^ The most frequent adverse events included injection-site pain (2.6%), headache (2.2%), and injection-site erythema (1.8%); injection-site reactions were mild and nonserious.^25^ Four participants discontinued because of adverse events considered unrelated to study treatment.^25^

Immunogenicity was also low. Among 2,104 participants across five Phase 1–3 GL trials who received up to three trenibotE treatments at doses of 100–750 U, only one participant (<0.1%) developed treatment-emergent binding antibodies, and no neutralizing antibodies were detected.^26^ The absence of neutralizing antibodies over the limited treatment cycles evaluated to date is reassuring; however, the available evidence is insufficient to establish a low risk of long-term immunogenicity or exclude potential cross-reactivity. Furthermore, findings from multiscale computational immunogenicity modeling suggest that BoNT/E may not represent a lower-risk alternative to BoNT/A, but rather a serotype with potentially greater immunogenic potential.^27^ Larger clinical studies with longer follow-up and repeated treatment cycles are needed to better characterize the immunogenicity profile.

### Potential role in aesthetic practice

There is limited published evidence characterizing patients who are reluctant to pursue neuromodulator treatment and who may therefore be particularly receptive to a short-acting option. However, consumer market research suggests that concerns regarding commitment to a long-lasting result, an unnatural appearance, adverse effects, and safety may represent important barriers to aesthetic treatment.^2^^,^^4^ Notably, nearly 9 in 10 patients expressed interest in previewing an aesthetic outcome before committing to treatment.^4^ TrenibotE may offer a lower-commitment entry point for toxin-hesitant patients seeking improvement in GL, particularly those concerned about unnatural or unsatisfactory outcomes with currently available BoNT/A products. Its shorter duration of effect may help reduce this treatment barrier before patients consider longer-lasting neurotoxin options. Even among patients already familiar with neuromodulator treatment, the rapid onset of trenibotE may be advantageous when treatment is sought shortly before a special event or when a faster visible response is desired. In addition, its shorter duration may also provide greater flexibility for patients whose aesthetic preferences change over time or when injection patterns and dosing are still being optimized before transition to a longer-acting toxin. Further patient-centered studies are needed to better define these potential clinical applications, identify the patients most likely to benefit, and evaluate patient satisfaction and treatment preferences.

Off-label use of neuromodulators in facial aesthetics is common in clinical practice, raising the possibility of trenibotE use beyond the procerus and corrugator muscles. Potential areas of interest include muscles in which unwanted weakness may carry greater functional or aesthetic consequences, such as the depressor anguli oris, as well as larger muscles such as the masseter, where treatment effects may take longer to become apparent. However, the optimal dosing, diffusion characteristics, efficacy, and safety of trenibotE in these off-label facial indications remain to be established.

Comprehensive immunogenicity assessments across clinical trials found no apparent effect of antidrug antibody formation on the safety or efficacy of trenibotE.^26^ Because BoNT/E is antigenically distinct from BoNT/A, trenibotE warrants investigation as a potential option for patients with secondary nonresponse associated with BoNT/A-neutralizing antibodies. However, multiscale computational immunogenicity modeling indicates that BoNT/E may have a relatively epitope-dense and structurally accessible antigenic profile, with the potential for cross-reactivity in individuals previously exposed to BoNT/A.^27^ Future clinical studies are needed to evaluate the potential role of BoNT/E in patients with secondary treatment failure attributable to neutralizing antibodies against BoNT/A.

### Knowledge gaps and future directions

The current evidence base has several important limitations. Although the Phase 2b study and one pivotal Phase 3 trial have been fully published, evidence from the second Phase 3 trial, the integrated immunogenicity analysis, and the sequential-treatment study is currently available only in conference abstract form, limiting the completeness of methodological appraisal and the overall level of evidence. In addition, the relatively limited number of treatment cycles reported in published studies restricts assessment of the durability of efficacy and the long-term safety and immunogenicity of repeated BoNT/E exposure. This is particularly relevant because neutralizing antibody development may emerge only after cumulative or prolonged treatment exposure. The predominance of industry-sponsored studies should also be considered when interpreting the available data, particularly in the absence of independent replication. Furthermore, no active-comparator trials have directly evaluated trenibotE against established BoNT/A formulations, limiting conclusions regarding relative efficacy, patient satisfaction, adverse-event profiles, and immunogenicity.

Published clinical studies have focused predominantly on GL, limiting generalizability to other facial muscles and aesthetic indications, and study populations have consisted largely of women and predominantly White participants, potentially limiting generalizability across more diverse racial and ethnic groups. Future studies should therefore include larger and more ethnically diverse populations to better characterize the efficacy, safety, and patient-reported outcomes of trenibotE across different demographic groups. Given its distinct dosing and duration profile, further studies should also characterize its diffusion properties and establish appropriate dosing, efficacy, and safety across additional facial muscles. In particular, studies in larger muscles such as the masseter are needed to determine optimal dosing, time to clinical response, and duration of effect.

From a pharmacologic perspective, longer posttreatment follow-up would be valuable to better characterize the duration and recovery profile of trenibotE. Although its short duration of action may be particularly appealing to patients who are hesitant to initiate neuromodulator treatment, evaluation across multiple treatment cycles with higher cumulative doses is also clinically important to assess the consistency of response, safety, immunogenicity, and patient experience over time. Future research could also examine whether satisfaction with trenibotE treatment encourages patients to subsequently consider longer-lasting neurotoxin options.

Current evidence is insufficient to establish dose equivalence between trenibotE and onabotA. Preclinical and head-to-head clinical studies directly comparing trenibotE with onabotA would therefore be valuable to better define relative potency, diffusion, efficacy, onset, duration, and safety, and to inform the potential use of trenibotE across indications currently treated with onabotA.

## Conclusion

TrenibotE is a rapid-onset, short-acting neuromodulator with demonstrated efficacy, high patient satisfaction, and a favorable safety profile for GL. In clinical practice, rather than competing primarily on duration, trenibotE introduces a distinct temporal treatment profile that may expand the ability to individualize neuromodulator therapy according to desired onset, duration, and treatment commitment. Its shorter duration may provide a lower-commitment option for toxin-naive or hesitant patients and expand opportunities for individualized treatment. Sequential administration with onabotA has also been shown to be well tolerated without apparent loss of efficacy or development of neutralizing antibodies. Future studies are needed to characterize repeated treatment cycles, diffusion, relative potency compared with BoNT/A, and its use beyond the glabellar complex. Overall, trenibotE may broaden the ability to individualize neuromodulator treatment according to patient preferences, desired onset, and duration of effect.

## Funding source

None.

## Ethical approval

Not Applicable.

## Data availability statement

Data sharing is not applicable to this article.

## Declaration of competing interest

Dr J Carruthers is a consultant and researcher for Allergan Aesthetics, and Dr A Michon is a consultant for Allergan Aesthetics. Dr A Michon is also a Guest Editor for the VSI: Injections on JPRAS Open and was not involved in the Editorial or Review process for this manuscript. The authors otherwise declare no competing interests relevant to this manuscript.
